# Transcriptomic and genomic analysis provides new insights in molecular and genetic processes involved in zucchini ZYMV tolerance

**DOI:** 10.1186/s12864-022-08596-4

**Published:** 2022-05-16

**Authors:** C. G. Amoroso, G. Andolfo, C. Capuozzo, A. Di Donato, C. Martinez, L. Tomassoli, M. R. Ercolano

**Affiliations:** 1 Department of Agricultural Science, University of Naples “Federico II”, Portici, NA Naples, Italy; 2 Department of Biology and Geology, Research Centers CIAIMBITAL and CeiA3, University of Almería, 04120 Almería, Spain; 3 Consiglio Per La Ricerca in Agricoltura e l’Analisi Dell’Economia Agraria, Research Centre of Plant Control and Certification, Rome, Italy

**Keywords:** Cucurbita pepo, RNA-seq, Transcriptome, Molecular markers, Plant recovery, Plant virus

## Abstract

**Background:**

*Cucurbita pepo* is highly susceptible to Zucchini yellow mosaic virus (ZYMV) and the resistance found in several wild species cannot be considered as complete or broad-spectrum resistance. In this study, a source of tolerance introgressed in *C. pepo* (381e) from *C. moschata,* in True French (TF) background, was investigated 12 days post-inoculation (DPI) at transcriptomic and genomic levels.

**Results:**

The comparative RNA-sequencing (RNA-Seq) of TF (susceptible to ZYMV) and 381e (tolerant to ZYMV) allowed the evaluation of about 33,000 expressed transcripts and the identification of 146 differentially expressed genes (DEGs) in 381e, mainly involved in photosynthesis, transcription, cytoskeleton organization and callose synthesis. By contrast, the susceptible cultivar TF triggered oxidative processes related to response to biotic stimulus and activated key regulators of plant virus intercellular movement. In addition, the discovery of variants located in transcripts allowed the identification of two chromosome regions rich in Single Nucleotide Polymorphisms (SNPs), putatively introgressed from *C. moschata,* containing genes exclusively expressed in 381e.

**Conclusion:**

381e transcriptome analysis confirmed a global improvement of plant fitness by reducing the virus titer and movement. Furthermore, genes implicated in ZYMV tolerance in *C. moschata* introgressed regions were detected. Our work provides new insight into the plant virus recovery process and a better understanding of the molecular basis of 381e tolerance.

**Supplementary Information:**

The online version contains supplementary material available at 10.1186/s12864-022-08596-4.

## Background

*Cucurbita pepo* is a high polymorphic species, including economically important crops grown worldwide. This species is highly susceptible to Zucchini yellow mosaic virus (ZYMV), a quickly spread aphid-borne potyvirus, leading to leaf yellowing and deformations, stunting and fruit defects [1–3]. To date, no sources of resistance to ZYMV have been found in this species, though in many areas the pathogen represents one of the most severe threats limiting crop production.

A wide range of ZYMV resistant genetic resources was found in other *Cucurbita* species [4, 5]*.* Several *C. moschata* accessions carrying resistance genes to ZYMV were identified: Nigerian local carrying *Zym0* and *Zym4* resistance gene [6]; Menina and Bolina possessing a resistance gene named *Zym1* [7]; Soler accession containing the recessive gene *zym6* [8] and Otto cultivar carrying four genes [9].

The genetic inheritance of tolerance to ZYMV derived from Menina in the *C. pepo* background showed that *Zym1* could interact with minor genes able to modulate the defense response [6, 10]. Recently, Capuozzo et al. [11] investigated *Zym1* segregation in different *C. pepo* populations. Genetic analysis for tolerance to ZYMV at both the phenotypic and genotypic levels clearly indicated that a major gene, *Zym1*, is essential for a tolerance expression in the 381e genotype background. Still, putative additional genes with additive or epistatic effects may mitigate or delay the symptoms. Pachner et al. reported that the combined deployment of seven genes in *C. pepo* would be required for maximal expression of ZYMV tolerance [12]. The involvement of putative modifying minor genes made difficult the understanding of genetic control and the subsequent transfer of ZYMV tolerance in breeding lines. In addition, the tolerant cultivar 381e when challenged with ZYMV showed a symptomatic systemic infection 4 days after inoculation (DPI) and a plant recovery, characterized by the emergence of newly developing leaves, about 12 DPI that can hamper genetic analysis [10].

Cucurbit genomic analysis and comparative gene expression analysis emerged as very useful tools for overcoming difficulties related to the introgression of desirable traits and for dissecting the molecular basis of host tolerance [13–15]. A reference zucchini genome version 4.1 (BGV004370) was recently provided, and several next-generation sequencing technologies, such as RNA-sequencing (RNA-Seq), have been explored for *C. pepo* [16–19]. To date, RNA-seq technology provides new opportunities for mapping and quantifying transcripts by creating new chances for the comprehension of loci, genes, and pathways activated during plant-pathogen interaction.

The main goal of this work was to investigate zucchini tolerance to ZYMV through genome-wide transcriptional analysis of the susceptible cultivar True French (TF) and a tolerant derived cultivar 381e. The modulation of gene expression in virus-infected plants was assessed at 12 DPI to highlight key genes activated in the plant recovering process in the tolerant accession. In addition, genomic scanning was performed to identify the candidate genes involved in ZYMV tolerance in *C. moschata* introgressed regions.

## Results

### *C. pepo* transcriptional profile upon ZYMV inoculation

**Fig. 1:**
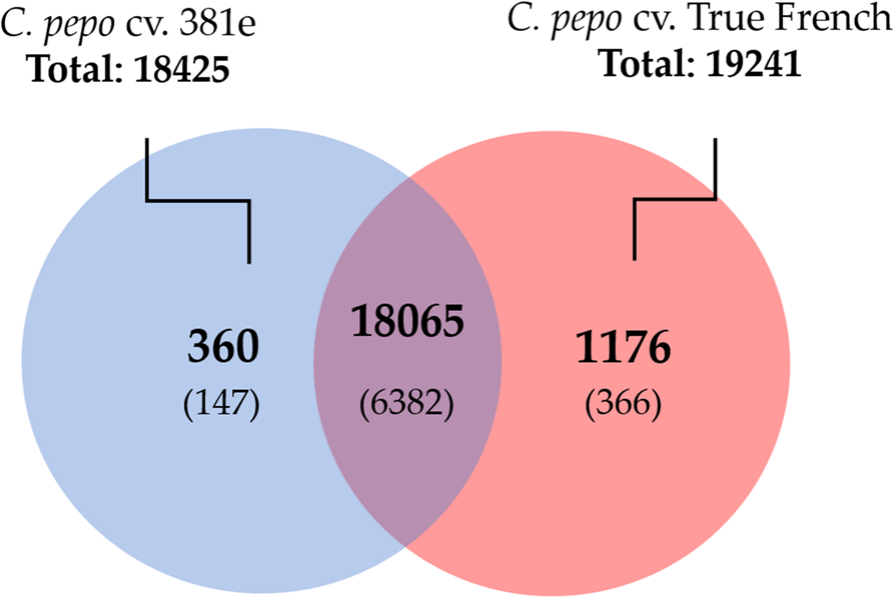
Transcriptional Profile. **A** Total Expressed Genes in *C. pepo* 381e (blue), TF (orange) and overlapped genes (violet). In parenthesis, differentially expressed genes for each area of the Venn diagram

The transcriptomic reprogramming of two zucchini isogenic cultivars (TF and derived ZYMV tolerant 381e) inoculated with ZYMV was evaluated by analyzing 33,388 genes expressed at 12 DPI. In TF-ZYMV interaction, transcriptome variation resulted in 19,241 expressed genes. As for the cultivar 381e, 18,425 transcripts were observed upon inoculation with ZYMV (Fig. 1). Exclusively expressed genes in 381e (360) were more than three times less than TF (1,176). Up to 6,382 transcripts out of 18,065 were differentially expressed in both cultivars, 147 were differentially expressed in 381e and 366 in TF (Fig. 1). Among the 3,681 up-regulated genes, 147 were exclusive of 381e, suggesting an important role in recovery during virus infection. DEGs identified in virus-challenged transcriptomes were further investigated to evaluate their implication in fundamental biological processes.

### Overview of cellular processes activated in the tolerant and susceptible cultivars

**Fig. 2:**
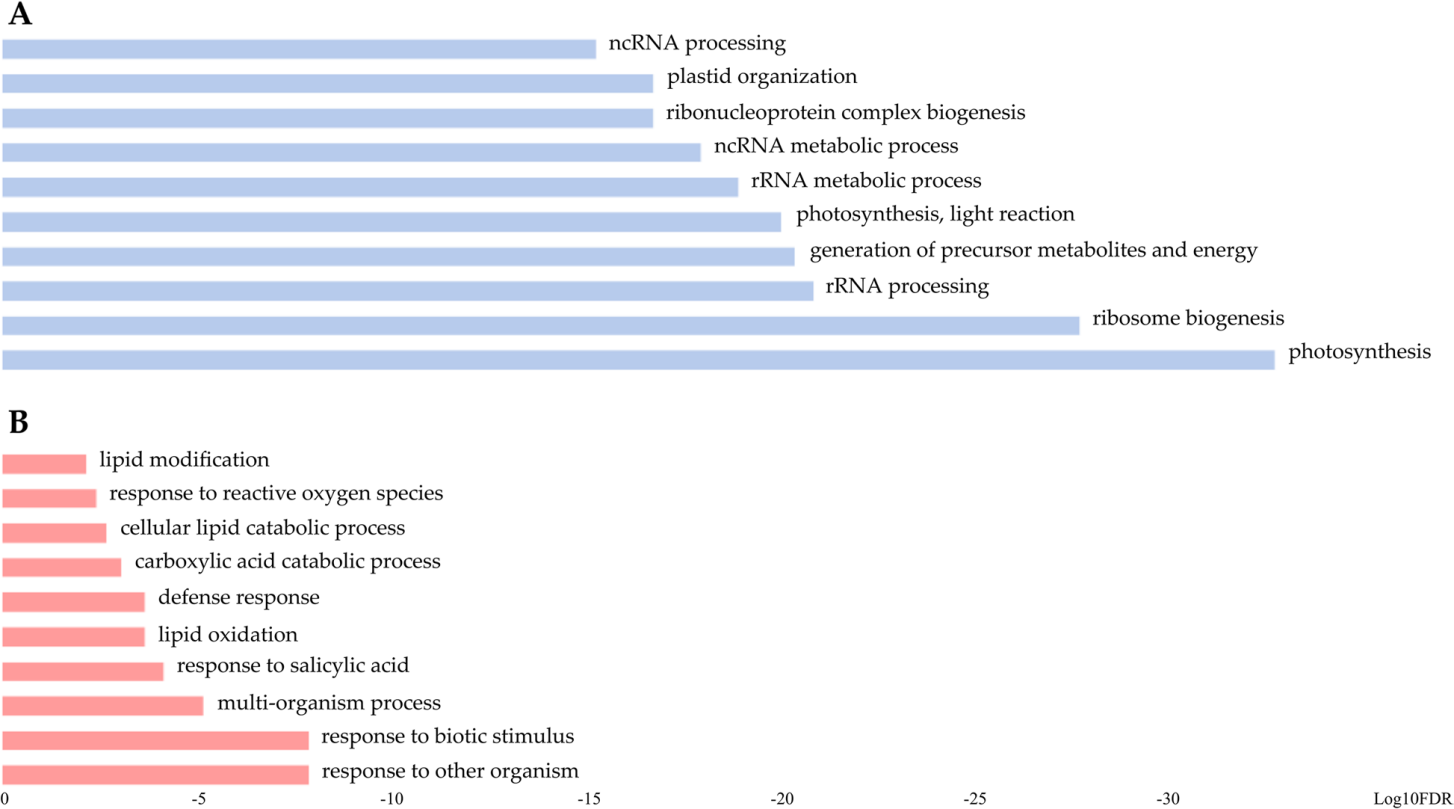
The top ten significantly enriched GO among all enriched GO child-terms (secondary level terms) starting from the root term biological process (GO:0,008,150)*.* **A** Enriched GO terms for up-regulated genes in 381e and **B**) Enriched GO terms for up-regulated genes in TF. The categories size is related to LOG_10_ FDR of enriched GOs

The identification of challenged processes after infection with ZYMV can help the understanding of tolerance response. According to the phenotypic recovery observed at 12 DPI, the tolerant cultivar 381e restored the main cellular activities (transcription, protein translation, photosynthesis, metabolites biosynthesis and the general cellular organization), which belonged to 139 GO term enriched categories [20]. Up-regulated genes in 381e were enriched in GO categories “photosynthesis” including 136 genes, "generation of metabolite and energy" and "photosynthesis, light reaction" including 190 and 77 genes, respectively (Fig. 2A). Most of these genes had a high LogFC (Cup000018g017445.1, LogFC = 2,32) and could be involved in the ZYMV recovery process.

By contrast, the susceptible cultivar TF showed 32 overrepresented GO terms, mainly involving receptors for biotic stress response, enzymes for ROS production, peptide degradation and lipid oxidation (Fig. 2B).

The plant vegetative restoring in 381e is promoted by increased photosynthetic activity through the up-regulation of genes involved in the formation of the photosystems (i.e., Cup000001g001195.1), in the electron transport chain and membrane transporters. In addition, genes directly associated with mitochondrial activity, glycolysis process (PEP, Acetyl-CoA, GAPDH, Hexokinaes), phospholipids synthesis (CDP-alcohol phosphatidyltransferase) and shikimic acid network synthesis were strongly up-regulated in 381e, suggesting an enhanced photo-respiratory activity. A strong up-regulation of genes involved in helicase activity, including DEAD-box ATP-dependent RNA helicases, DNA replication licensing factor, such as the exclusively expressed Cup000013g012986.1, and two DNA-binding protein (Cup000085g037850.1 and Cup000022g020052.1) was observed in 381e. Moreover, several Heat Shock Proteins (HSPs) proteins were found differentially regulated in both cultivars. In particular, 381e activated HSP 90–5 (Cup001195g045603.1), potentially involved in the transport of newly synthesized proteins from ribosomes to chloroplasts, and HSP-70 (Cup000010g010288.1), involved in proteins trafficking through the chloroplast membrane to stroma in cooperation with 14–3-3 protein (Cup000060g034360.1), that in this study also resulted overexpressed [21]. Furthermore, seven aquaporin membrane transporters (i.e. Cup000679g045055.1) resulted strongly up-regulated in 381e(LogFC > 1).

By contrast, TF showed a high activation of genes involved in the chloroplast architecture (i.e., Cup000003g002638.1) and oxygen scavenging, such as two L-ascorbate peroxidases (Cup000004g003828.1 and Cup000052g032798.1) belonging to "reactive oxygen species" GO term (Fig. 2B). Furthermore, we also found the up-regulation of catalases and superoxide dismutases (i.e., Cup000032g025333.1) and a protein ridA (Cup000182g042991.1) involved in response to BCAT3 (Branched-chain-amino-acid aminotransferase 3) that is produced in response to stress. Interestingly, we found the up-regulation of several cytochromes type p450 associated with the endoplasmic reticulum and involved in the metabolism of chemical compounds extraneous to the organism. A cytochrome CYP82C2 (Cup000001g001147.1), directly involved in the jasmonic acid network synthesis, was highly expressed, suggesting that the plant defense system was still alerted [22]. In addition, two Synaptotagmins and SNAP receptors (Cup000004g004336.1, Cup000029g023659.1, Cup000011g011417.1), belonging to the *"*Response to other organisms" GO term, resulted up-regulated and exclusively expressed in TF (Fig. 2B).

In our study, 29 serine/threonine protein kinases resulted up-regulated in 381e while 42 were up-regulated in TF. Among these, 22 were localized on the plasma membrane. In addition, TF differentially expressed several genes involved in the cell wall degradation (i.e., pectinesterases, beta-glucosidases and xyloglucan endo-transglycosylases), indicating the collapse of the primaries structure of the plants. On the other hand, it is interesting to note that genes involved in cytoskeleton organization (Cup000006g006297.1), in glycosaminoglycan biosynthesis (Cup000019g017783.1) as well as two Rab GTPases (Cup000067g035924.1 and Cup000038g027690.1) and a gene having a crucial role in the callose synthesis (Cup000021g019191.1) resulted exclusively up-regulated in 381e.

### Genomic localization of transcript variants

In order to identify putative genomic regions introgressed into C. *pepo* from C. *moschata* cv. Menina, we performed a variant calling in both the near-isogenic cultivars, TF and 381e was conducted.

**Fig. 3:**
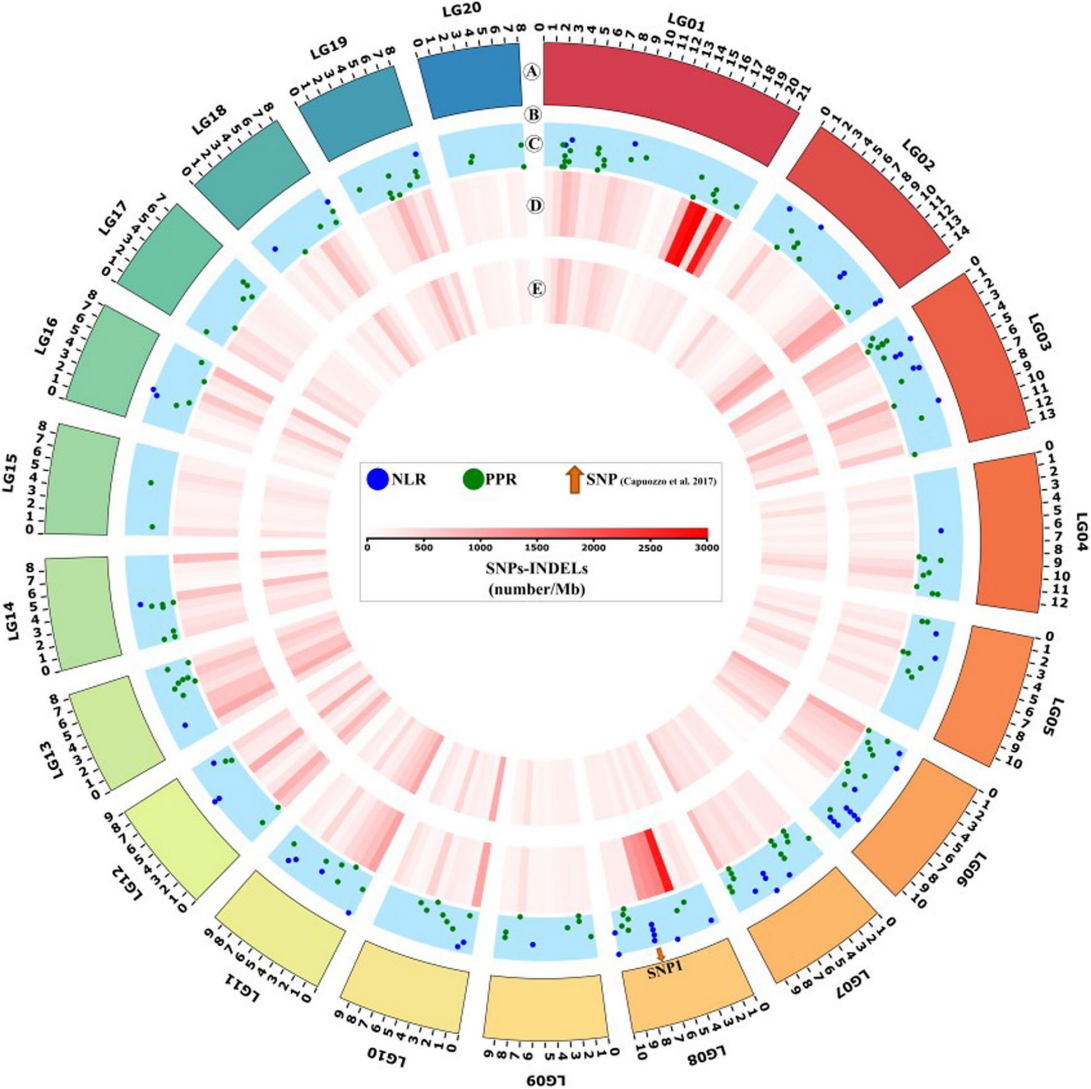
Circos plot integrating the genomic positions of ZYMV-related markers, pathogen recognition genes and small variants (SNP, Single Nucleotide Polymorphisms and InDel: insertion/deletion). Inset legend provides information for the data rings. Track A denotes the 20 pseudochromosomes (LG, linkage group) of *Cucurbita pepo*. The length of each circle segment represents the size of pseudochromosomes expressed in megabases (Mb). An orange arrow indicated the marker associated with ZYMV tolerance identified by Capuozzo et al. [11]. Track B shows, as a scatterplot, the genomic position of nucleotide-binding domain leucine-rich repeat containing receptors (NLR; blue spots) and receptor-like proteins and receptor-like kinase (PPR; green spots), annotated by Andolfo et al. [19]. Tracks C and D show the variants density, represented as heat maps (number/Mb) in *C. pepo* 381e and *C. pepo* TF cultivars, respectively

**Fig. 4:**
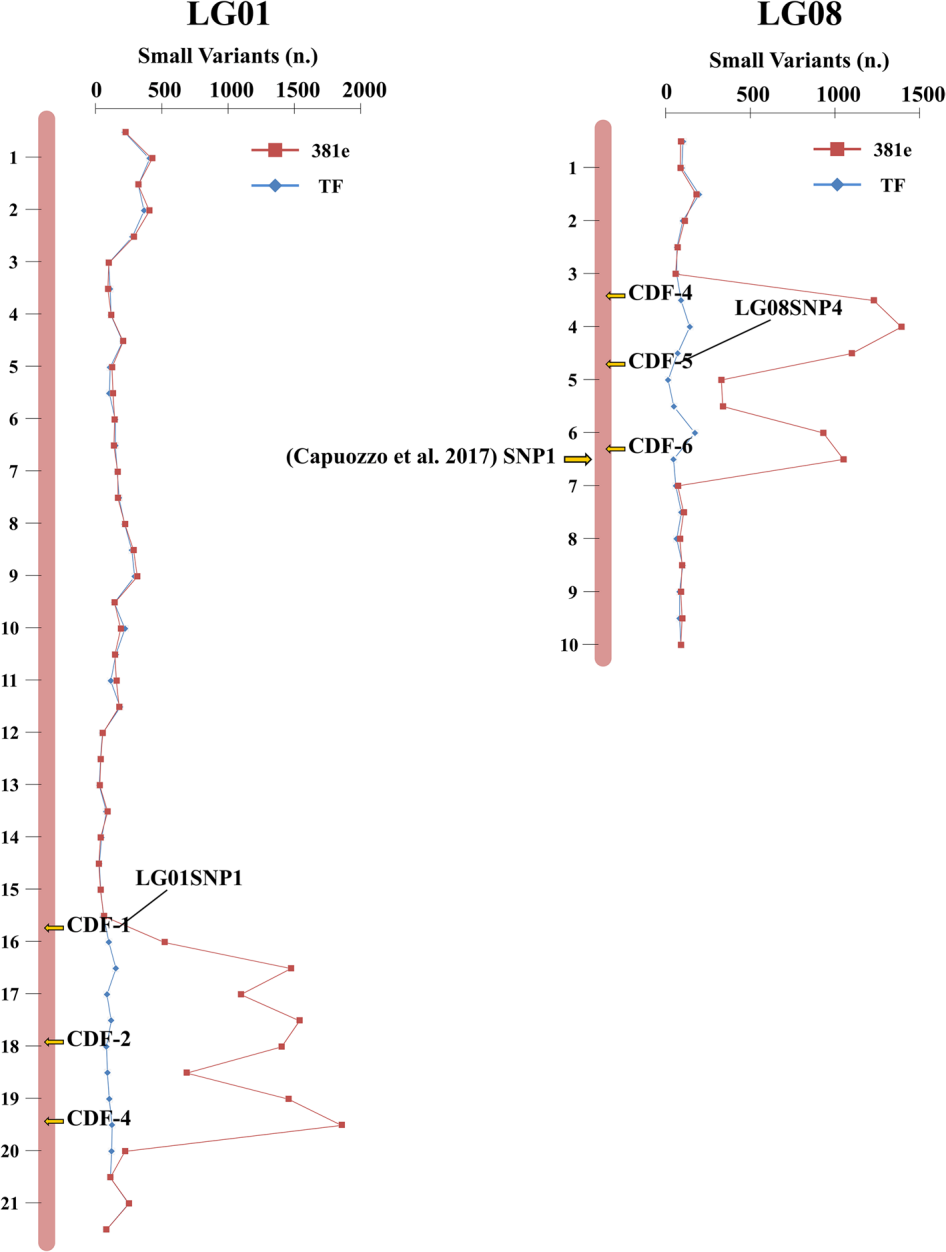
High-resolution map integrating genomic and genetic marker informations. The genomic position of six coding DNA fragments (CDFs) and two CAPS markers (LG01SNP1 and LG08SNP4) is indicated. The distribution of small variants along 1 and 8 pseudochromosomes is reported in red (381e) and in blue (TF)

A total of 150,605 high-quality small variants, including SNPs (Single Nucleotide Polymorphisms) and InDels (insertions/insertions/deletions), were mapped to the *C. pepo* BGV004370 reference genome (Fig. 3, Additional Table 1) [16]. The number of common small variants in 381e and TF was normalized with respect to the LG (Linkage Group) length (Fig. 3). In 381e, on average, eight variants were mapped for expressed locus, on LGs 01 and 08, while in TF two variants per transcript were found on such LGs (Additional Table 2). About 30% of small variants identified in 381e mapped on two LGs, while in TF the percentage of variants on the same LGs was close to 13% (Additional Tables 1 and 2 and Fig. 3). A close-up view of LGs 01 and 08 of the two *C. pepo* cultivars showed that the 20% (16,891) of small variants identified in 381e were concentrated in two short regions of LGs 01 and 08, while in TF less than 3% (1,410) were mapped in the same areas (Fig. 4). In 381e, over 65% (10,321) of small variants identified on LG 01 were located on a genomic region of about 4 Mb, while on LG 08, the second putative introgression region of 3,5 Mb showed 82% (6,570) of total variants. Interestingly, Capuozzo et al. mapped, on the same LG 08 region, the marker (SNP1) associated with ZYMV tolerance in 381e (Fig. 4 and Additional Table 2) [11].

### Variants validation and marker-phenotype correlation

Molecular validation was performed to confirm the DNA variants identified on putative introgression regions located on LG 01 and LG 08. A total of six coding DNA fragments (CDFs), located on two genomic regions inherited from *C. moschata,* were sequenced and analyzed (Fig. 4). About 70 SNPs, annotated into the six CDFs, were confirmed from Sanger sequencing (Additional Table 3). Two high confidence variants (LG08SNP4 and LG01SNP1) were converted in CAPS markers (Additional Table 3; Additional Fig. 1) to conduct a marker-phenotype correlation analysis. LG08SNP4 was physically close (~ 2 Mb) to the previously mapped "SNP1" and exhibited a very high correlation coefficient (Pearson test *p*-value < 0,01; *r* = 0,991) with phenotypic data [11]. In particular, marker correlation with ZYMV-resistance or susceptibility traits was in accordance for 73 genotypes out of 82 genotypes analyzed. A good correlation coefficient with phenotypic results was displayed also by LG01SNP1 (*r* = 0,827; Pearson test *p*-value < 0,05). In addition, an interesting correlation (*r* = 0,895; Pearson test *p*-value < 0,05) was identified between the markers LG01SNP1 and LG08SNP4, located on chromosome 1 and 8, respectively.

## Discussion

From its first report in the late 1970s, ZYMV continued its worldwide expansion, causing several yield losses in cucurbits. This work explored the molecular basis of ZYMV tolerance identified in *Cucurbita moschata* cv Menina, analyzing the derived *C. pepo* tolerant cv 381e and its susceptible counterpart TF through an integrated transcriptomic and genomic experiment. Though *C. moschata* is sparingly cross-fertile with *C. pepo*, the gene expression may be hampered in new genomic background [6]. In addition, approximately 12 days after ZYMV inoculation, 381e plants showed a vegetative recovery [10], while damaged TF plants displayed yellowing of foliage, internodes shortening and leaf deformations.

In our study, an opposite response to infection was clearly observed at 12 DPI, in terms of expressed and differentially regulated genes between the two analyzed genotypes. The most represented GO categories in 381e emphasized the recovery process mediated by increased photosynthetic activity and general cell restoring. For instance, in "photosynthesis", "generation of precursor metabolites and energy" and "photosynthesis, light reaction" GO terms were included genes with high LogFC (Cup000018g017445.1, LogFC = 2,32; Cup000025g021749.1, LogFC = 1,83 and Cup000010g010661.1, LogFC = 1,02). By contrast, among down-regulated genes exclusively expressed in TF, Cup000011g011417.1 (LogFC = -1,19) belonged to the GO term "Response to other organisms" and could be involved in TF-ZYMV susceptibility.

ZYMV virus usually takes about nine days to infect the plant systemically through the phloem [23] and the early systemic infection of 381e, about 4 DPI, could prompt a plant recovery. At the initial stage of infection, the plant moderates its immune response and general fitness, triggering a decrease in symptoms of viral infection [24]. The 381e physiological perturbations imposed various constraints to face virus, limiting the synthesis of host proteins essential for replication and movement. Tolerant plants react to virus infection through changes in physiological and developmental processes to reduce the synthesis of factors required for virus multiplication [25–27]. We found several DEAD-box RNA helicases and DNA helicases, such as Cup000013g012986.1, up-regulated in 381e*.* In particular**,** Cup000013g012986.1 belonged to "DNA unwinding during replication" GO category (supplementary material 4). DEAD-box RNA helicases can function as viral RNA sensors or effectors by blocking virus replication. RH30 (Dead-box helicase) was required to restrict *Bushy Stunt Virus* (TBSV) replication in tomato [28]. Moreover, RNA and DNA helicases can also positively affect stress tolerance [29]. A general gene down-regulation could be useful to impede virus movement and replication, leading to a less virus titer and promoting a consequent plant recovery [23]. By contrast, the up-regulation of factors correlated with transcription processes observed in TF plants may facilitate the use of the host nuclear factors for ZYMV replication [30].

In our experiment, the tolerant cultivar 381e showed an increased photosynthetic and photo-respiratory activity promoting recovery and symptoms alleviation. The response to viral infection in 381e required a global host transcriptome reprogramming to avoid the developing of more severe symptoms. Indeed, the up-regulation of genes involved in energy production and cell repairing was observed at 12 DPI. Our results were in agreement with Nováková et al. [31], in which the partially resistant *C.pepo* cv Jaguar showed a strong up-regulation of several proteins involved in photosystems activities 15 days after ZYMV inoculation [31]. Interestingly, we found a high up-regulated gene coding for an *HSP90.5* (Cup001195g045603.1) with a LogFC of 4.6 exclusively expressed in 381e (GO:0045037, supplementary material 4)*.* Proper control of *HSP90.5* expression was required for plant growth and development and was essential for the formation of chloroplast thylakoids and the regulation of receptor proteins involved in plant immunity [32–34]. In addition, the overexpressed *HSP70* (Cup000010g010288.1) cooperates with the overexpressed 14–3-3 chaperone (Cup000060g034360.1) to keep the chloroplast or mitochondrial precursor proteins in an unfolded state and to translocate them through plasmodesmata [35, 36]. By contrast, the TF photosynthetic system was affected by the down-regulation of the genes involved in the formation of chloroplasts and thylakoids.

Symptom recovery is generally accompanied by the activation of pathogen receptor genes and other defense-related genes during the plant-pathogen interaction [23, 37, 38]. In particular, we found a resistance protein (Cup000003g003423.1, CNL), annotated by Andolfo et al., [19] and located on linkage group 8 scaffold 3, that was up-regulated and privately expressed (LogFC = 1.51) in 381e [19, 39, 40]. It is interesting to note that this transcript carried a variant leading to functional modification. In our study, numerous genes codifying for serine-threonine kinases proteins were found to be differentially expressed in both cultivars. In particular, seven proteins showed a high homology to well-characterised R-genes as identified by Andolfo et al. (2017) and may play a central role in signalling during zucchini-ZYMV interaction (Supplementary Table 6).

Furthermore, SNP1 was localized in the coding sequence of an RNA helicase. Deleterious effects on viral ToMV infectivity after the loss of function of helicase genes and in response to other stresses have been described [41, 42].

A serine-threonine kinase (Cup000024g021133.1), belonging to the RIO protein family, was down-regulated and privately expressed in TF. This gene was located on linkage group one (scaffold 24), corresponding to one of the regions putatively introgressed from Menina in 381e. These proteins are generally involved in the biogenesis of small ribosomal subunits and were found to interact with the *Tomato Mosaic Virus* (ToMV) movement protein (MP) to promote virus movement [43, 44].

The ZYMV virus enters the cells through wounds or introduced by aphids and moves from cell to cell through the plasmodesma to reach the phloem, where it induces systemic infections. A delicate equilibrium between RNA silencing and virus counter-defense responses in recovered leaves may help in maintaining the viral levels below the threshold required for the symptom induction. In TF, we found the up-regulation of the *CYP82C2* (Cup000001g001147.1) gene, which in Arabidopsis has a role in JA-induced defense genes [45]. In addition, we found two synaptotagmins (*SYTA*) exclusively expressed in TF. These proteins are key regulators of plant virus intercellular movement, able to promote the movement of the proteins through the cells and to regulate endocytosis and protein-mediated trafficking through plasmodesmata [46, 47].

In 381e, the up-regulation of genes involved in cytoskeleton organization, vesicular trafficking and callose deposition, such as Cup000021g019191.1 (LogFC = 1.59), could enhance plant tolerance by reducing the virus spread from cell to cell through the regulation of plasmodesmal permeability [48, 49]. By contrast, the up-regulation of genes involved in cell wall degradation (such as the pectinesterases) was observed in TF.

Since 381e and TF were two near-isogenic cultivars, comparing them at genome level allowed us to identify two regions enriched in SNPs in the tolerant cultivar 381e. These regions could be considered introgressed from Menina and therefore putatively involved in 381e tolerance.

The tolerance level of 381e to ZYMV was not as high as in *C. moschata* cv Menina, and some other genes could be involved in the resistance regulation [6, 50]. A marker located on chromosome 8, showing 90% co-segregation with tolerant phenotypes, was previously identified in an F2 population derived from the cross between 381e and TF [11]. The correlation of LG08SNP4 with resistance traits in the F2 population (381e x TF) was also very high, supporting the finding of Capuozzo et al., [11]. Furthermore, the lower correlation of LG01SNP1 with tolerant phenotypes suggested that genes with minor effects on the virus tolerance expression could be located on LG 01. Our RNA-seq analysis allowed us to identify candidate genes that could be involved in 381e-ZYMV tolerance. However, other studies should be carried out to define better which genes are responsible for tolerance. The identification of introgressed regions emerging from our study is essential for discovering new genetic markers associated with ZYMV tolerance. Further experiments could be done to narrow down the region and characterize differential expressed genes by biotechnological approaches.

## Conclusion

In conclusion, the network of expressed genes at 12 days after virus infection in 381e revealed the recovery of physiological processes. In particular, we found the differential regulation of genes involved in the photorespiration process (Cup000018g017445.1, Cup000025g021749.1 and Cup000010g010661.1), in transport (Cup000060g034360.1) and possibly, affecting virus replication and movements (Cup000013g012986.1, Cup000003g003423.1). Furthermore, two introgressed regions in 381e from Menina, containing NLR genes, serine-threonine and DEAD-box RNA helicases, could be involved in tolerance to ZYMV. In particular, on linkage group eight might be located a major gene responsible for tolerance and on linkage group one a minor gene with an additive effect that complements the major gene's contribution. Further experiments should be performed to fine mapping contributing loci and to functional characterize differential expressed genes.

## Materials and methods

### Plant material and ZYMV-inoculation

**Fig. 5:**
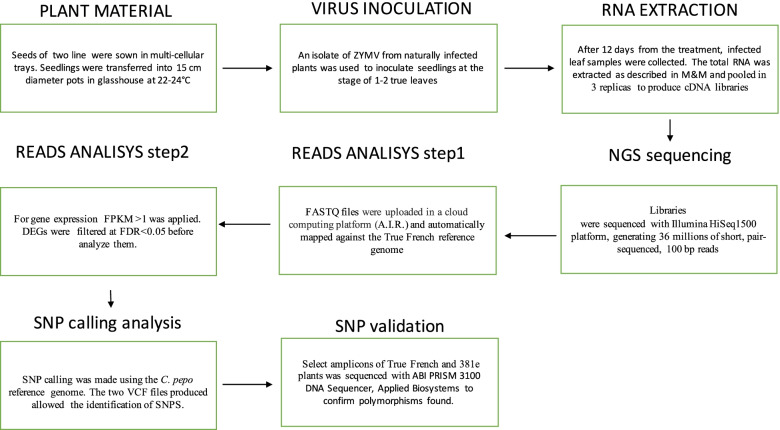
Graphical experimental schema

Two zucchini near-isogenic cultivars, TF (susceptible to ZYMV) and 381e (tolerant to ZYMV), were kindly provided by La Semiorto Sementi. These cultivars were used for an RNA-seq experiment and DNA sequencing, following the scheme reported in Fig. 5. The latter cultivar has been derived from six generations of backcrossing to TF, selected for tolerance, and followed by four successive generations of self-pollination [10]. Forty seeds of each cultivar were sown one per pot in multi-cellular trays consisting of 4 cm diameter pots filled with peat. After the sowing, seedlings were transferred into pots with diameters of 15 cm and were grown under glasshouse condition at 22–24 °C using supplement lighting to maintain 12 h photoperiod at the Research Centre for Plant Pathology (CRA-PAV) in Rome. An identification number was assigned to each plant. In our experiment, we used an isolate of ZYMV from a naturally infected field-grown summer squash plant. The isolate caused the typical symptoms of ZYMV disease, including yellow mosaic, vein banding, blistering and leaves malformations. Symptomatic leaves of artificially infected zucchini plants, kept in greenhouse for symptom development, were crushed, and the raw juice was extracted at a ratio of 1:10 w/v in 0.1 M phosphate buffer pH 7.2. Seedlings at stage of 1–2 leaves, sprinkled with the abrasive powder "celite," were inoculated with approximately 20 μl of the diluted extract and subsequently washed with distilled water. Non-inoculated plants were used as control.

### Sample collection and nucleic acid isolation

Twelve days after the treatment, infected zucchini leaf samples of three independent replicates were collected. Leaves were removed from the plants, weighed and immediately frozen in liquid nitrogen and stored at − 80 °C. RNA was isolated using the RNAeasy Plant Kit according to the manual instructions (Qiagen Valencia, USA). The concentration of RNA samples was determined using a NanoDrop ND-1000 spectrophotometer (Nano-Drop Technologies, Wilmington, DE, USA) and an Agilent 2100 Bioanalyzer (Agilent Technologies). RNA integrity was checked by horizontal electrophoresis on a 1.2% (w/v) agarose gel. Two μg of each sample were prepared with 20 μl of 10 X RNA Loading Buffer composed of 400 μl Formamide, 10 μl 37% formaldehyde, 2 μl loading buffer 10X (50% glycerol, 0.25% w/v bromophenol blue, 0.25% w/v xylene cyanol; Sigma) and 1 μl of 10 mg/μl SYBR Safe DNA Gel Stain (Invitrogen). Gel visualization was performed using UV light (UV Gel Doc BIORAD).

Total purified RNA of three independent replicas was converted to cDNA libraries (QuantiTect Reverse Transcription Kit, Qiagen) and sequenced on Illumina HiSeq1500 platform at the LabMedMolGe (Laboratory of Molecular Medicine and Genomics Department of Medicine and Surgery, University of Salerno, following a paired-end sequencing (2 × 100 bp).

### RNA-Sequencing data analysis

The raw reads obtained for each sample, following Illumina sequencing, were analyzed using the online platform A.I.R. (https://transcriptomics.sequentiabiotech.com), containing algorithms and software packages described in Vara et al. [51]. After a quality check (adapter removal and trimming of low-quality reads), the cleaned reads were mapped against the *C. pepo* reference genome, and genes with an FPKM value > 1 were considered expressed. The number of reads for each sample before and after the quality check, the mean GC content and the sequence length are reported in supplementary materials (Supplementary Table 5). The read counts obtained were analyzed using the DESeq2 algorithm to identify differentially expressed genes (DEGs). Genes with an FDR (using Benjamini–Hochberg correction method) < 0.05 were considered DEGs and used for further analyses. DEGs with positive or negative logFC values were classified as upregulated (logFC > 0) or downregulated (logFC < 0), respectively. Gene Ontology Enrichment Analysis on DEGs was performed using AgriGO version 2.0 [52]. The *C.pepo* reference genome version 4.1 (BGV004370) and relative gene annotation used for the analysis were reported by Montero‐Pau et al. and Andolfo et al., respectively [16, 19].

### Variant calling and genome plot construction

A SNP calling analysis was performed to detect SNPs and InDel, on the transcripts of the susceptible TF and tolerant 381e accessions, compared to *Cucurbita pepo* reference genome version 4.1 (BGV004370) [16]. Position of SNPs and InDel within the aligned reads compared to the reference genome were identified using the pileup function in SAMtools v1.9 [53]. SNPs were filtered using a minimum SNP quality score (QUAL), mapping quality (MQ) and genotype quality (GQ) of 30, an allele frequency (AF) higher than 0.75 and a minimum and maximum read depths (coverage) set to 5 and 100, respectively. Finally, the circular multi-track plot was carried out using the “RCircos” R package [54].

### Validation of predicted variants

DNA was extracted from not infected leaves (control plants) of the 381e and TF using DNeasy Plant Mini Kit (Qiagen). DNA sample concentration was determined using a NanoDrop ND-1000 Spectrophotometer (Nano-Drop Technologies, Wilmington, DE, USA). In addition, the DNA of an F2 segregating population (381e x TF) obtained by Capuozzo et al. was also used for markers validation [11]. A pool of six coding DNA regions was selected to perform the molecular validation of the variants identified in 381e and TF. PCR was executed with 25 ng of genomic or complementary DNA, 10 pmol primers, 1 U of Taq DNA polymerase kit (Invitrogen, Carlsbad, CA, USA), 10 pmol dNTPs, and 2 mM MgCl2 in 25 μl reaction volumes. Amplification was performed using the following cycling conditions: 1 min at 94 °C, followed by 30 cycles of 1 min at 94 °C, 1 min 30 s at 60 °C and 2 min at 72 °C, with a final extension for 7 min at 72 °C. Amplicons were separated by electrophoresis on agarose gel (1.5%) and photographed by a GelDoc apparatus. Primers were designed with Primer3 (http://frodo.wi.mit.edu), with a length between 18 and 27 bp. The length of the amplified fragments ranged from 300 to 1,000 bp, and the Tm of the specific primers was 59 °C for all pairs of primers (Online Resource S4). Amplicons were sequenced using the BigDye Terminator Cycle Sequencing Kit (Applied Biosystems, Foster City, CA, USA) and run on automated DNA sequencers (ABI PRISM 3100 DNA Sequencer, Applied Biosystems). Sequence data deriving from *C. pepo* reference genome v 4.1 (BGV004370) were aligned with corresponding sequences originated from amplicons, using MUSCLE 3.6 [55]. In addition, two couples of primers were tested on a subset of the F2 segregating population (381e x TF) phenotyped by Capuozzo et al. [11]. Both Pearson and Spearman correlation coefficients were applied on marker-phenotype data.

## Supplementary Information

**Additional file 1: Table S1. **Number of short variants (SNV and InDel) identified in TF and 381e. For each C. pepo linkage group (LG) the length, percentage of expressed genes (EGs) and number of normalised variants are reported. **Table S2. **Number of short variants (SNV and InDel) identified in TF and 381e. For each C. pepo linkage group (LG) the length, percentage of expressed genes (EGs) and number of normalised variants are reported. **Table S3.** Validation of 6 polymorphic loci located on putative genomic regions introgressed from ‘Menina’. For each amplicon, locus name, primer and polymorphism information are reported. **Additional Table 4.** Protein function of gene annotation reported by Andolfo et al. [11]. **Additional Table 5.** Summary of the RNA-Seq data. Number of reads before and after the quality check, mean GC content and sequence length were reported for each biological replicate (BR). **Supplementary Table 6.** List of differentially expressed receptor-like serine/threonine-protein kinases that were identified and classified in Cucurbita pepo cv. True French by Andolfo et al., (2017). **Supplementary Table 7.** High confident UP and DOWN regulated genes (Up regulated with LogFC > 1 and Down regulated with LogFC < 1).

**Additional file 2: Additional Figure 1. **Gel electrophoretic separation of digested PCR products (left 381e, right TF); panel A: LG01SNP1; panel B: LG08SNP4. Relevant fragment sizes (bp) are denoted on the right side (1Kb plus). **Additional Figure 2. **Comparison of the ZYMV tolerant line 381e (left) and the susceptible True French (right) 12 days after ZYMV inoculation.

## Data Availability

The data set supporting the results of this article is available in the Sequence Read Archive (SRA) repository of NCBI under the GEO accession number: GSE159530, at link: https://www.ncbi.nlm.nih.gov/geo/query/acc.cgi?acc=GSE159530

## Abbreviations

ZYMV: Zucchini Yellow Mosaic Virus
DPI: Days Post Inoculation
DEGs: Differentially Expressed Genes
SYTA: Synaptotagmin
CNL: Coiled-Coil Nucleotide binding domain Leucine rich repeat
TF: True French
FDR: False Discovery Rate
GO: Gene Ontology
ROS: Reactive Oxygen Species
PEP: Phosphoenolpyruvate carboxylase
GAPDH: Glyceraldehyde 3-phosphate dehydrogenase
HSP: Heat Shock Protein
SNV: Single Nucleotide Variants
INDEL: Insertion and Deletion
LG: Linkage Group
NLR: Nucleotide-binding domain leucine-rich repeat containing receptors
PPR: Proteins and Receptor like Kinase
CDF: Coding DNA Fragments
