# Supplementary material for: Transcriptomic and genomic analysis provides new insights in molecular and genetic processes involved in zucchini ZYMV tolerance
Source: BMC Genomics. 2022 May 16;23:371. doi: 10.1186/s12864-022-08596-4 (PMC9109310; doi:10.1186/s12864-022-08596-4)
Supplement: Supplementary file 2 — Additional file 2: Additional Figure 1. Gel electrophoretic separation of digested PCR products (left 381e, right TF); panel A: LG01SNP1; panel B: LG08SNP4. Relevant fragment sizes (bp) are denoted on the right side (1Kb plus). Additional Figure 2. Comparison of the ZYMV tolerant line 381e (left) and the susceptible True French (right) 12 days after ZYMV inoculation. [file 12864_2022_8596_MOESM2_ESM.docx]

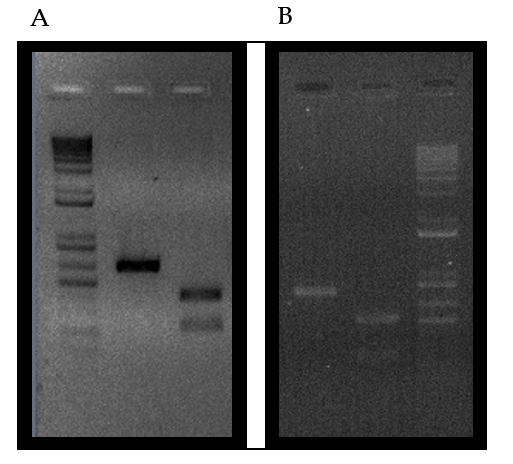


Additional Figure 1: Gel electrophoretic separation of digested PCR products (left 381e, right TF); panel A: LG01SNP1; panel B: LG08SNP4. Relevant fragment sizes (bp) are denoted on the right side (1Kb plus).


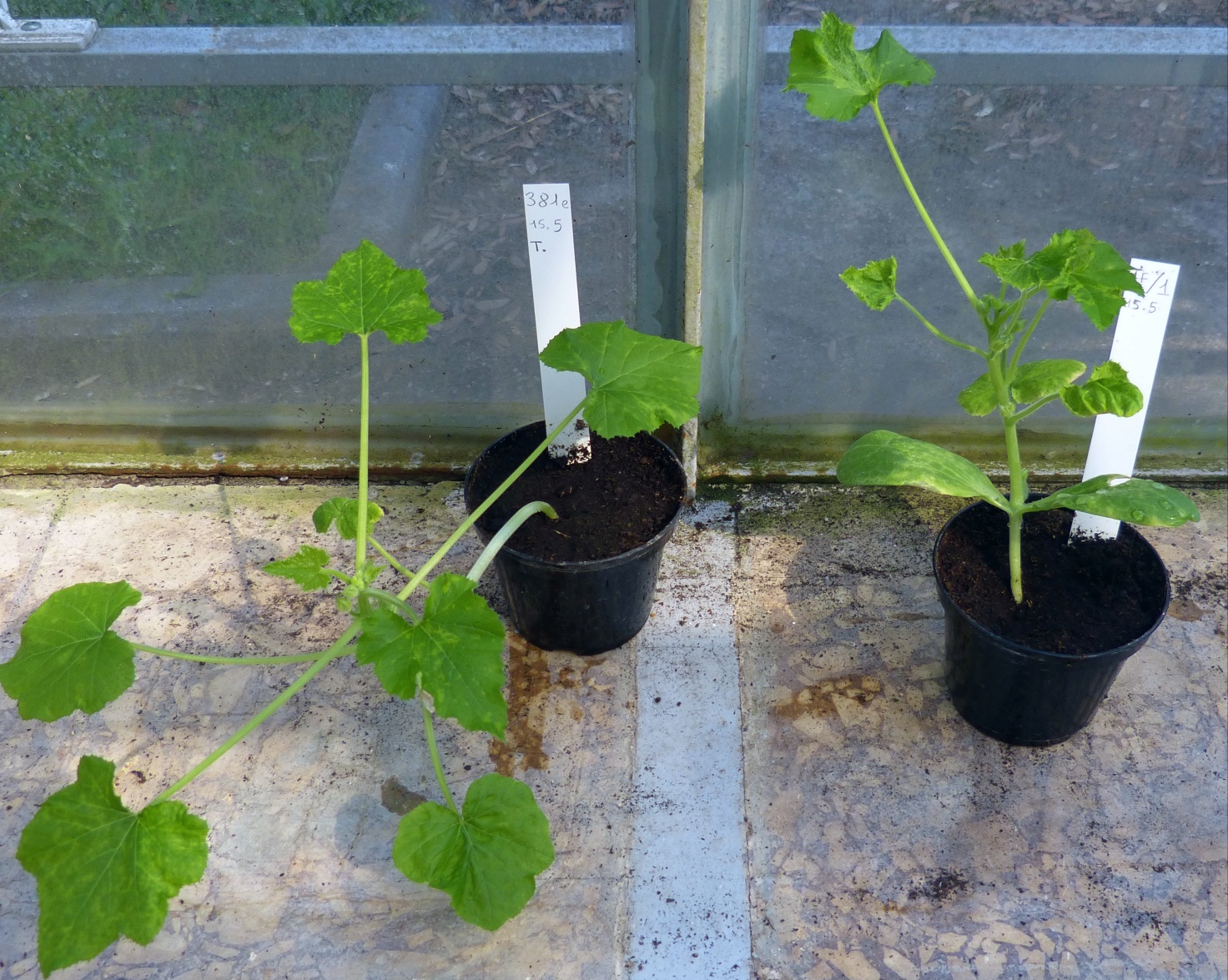


Additional Figure 2: Comparison of the ZYMV tolerant line 381e (left) and the susceptible True French (right) 12 days after ZYMV inoculation.
